# MYC dysfunction modulates stemness and tumorigenesis in breast cancer

**DOI:** 10.7150/ijbs.51458

**Published:** 2021-01-01

**Authors:** Yiqiu Liu, Chengjun Zhu, Lin Tang, Qin Chen, Nan Guan, Kun Xu, Xiaoxiang Guan

**Affiliations:** 1 Department of Oncology, The First Affiliated Hospital of Nanjing Medical University, 300 Guangzhou Road, Nanjing 210029, China.; 2 Department of Medical Oncology, Medical School of Nanjing University, Nanjing, 210002, China.; 3 College of Letters and Science, University of California, Los Angeles, 405 Hilgard Avenue, California, 90095, USA.

**Keywords:** MYC, cancer stem cells, breast cancer, tumorigenesis

## Abstract

As a transcription factor and proto-oncogene, MYC is known to be deregulated in a variety of tumors, including breast cancer. However, no consistent conclusion on the role and mechanism of MYC deregulation during breast cancer carcinogenesis has been formed. Here, we used the UALCAN, bc-GenExMiner, TCGA, cBioportal, STRING and Kaplan-Meier Plotter databases to explore the mRNA expression, prognosis, transcriptional profile changes, signal pathway rewiring and interaction with the cancer stem cells of MYC in breast cancer. We found that the expression of MYC varies in different subtypes of breast cancer, with relatively high frequency in TNBC. As a transcription factor, MYC not only participates in the rewiring of cancer signaling pathways, such as estrogen, WNT, NOTCH and other pathways, but also interacts with cancer stem cells. MYC is significantly positively correlated with breast cancer stem cell markers such as CD44, CD24, and ALDH1. Collectively, our results highlight that MYC plays an important regulatory role in the occurrence of breast cancer, and its amplification can be used as a predictor of diagnosis and prognosis. The interaction between MYC and cancer stem cells may play a crucial role in regulating the initiation and metastasis of breast cancer.

## Introduction

Based on the latest statistics from the American Cancer Society and the National Cancer Institute, breast cancer, with the highest prevalence and mortality, accounts for approximately 44% of all cancers among female 1. Despite the yearly increase in incidence, the five-year survival rate of breast cancer has improved from 79% in 1986 to 91% in 2014, excluding triple-negative breast cancer (TNBC) whose 5-year survival rate ranks lowest 2. Nevertheless, the gradually decreasing medium age of diagnosis and the increasing ratio of tumor metastasis among survivors with relatively limited treatment measures, including radiotherapy, chemotherapy and surgery, all demonstrate the fact that breast cancer is still an arduous challenge for researchers and patients, highlighting the urgency of discovering new diagnostic biomarkers and therapeutic targets 1, 3.

MYC is a transcription factor whose family includes c-myc, B-myc, L-myc, N-myc and s-myc 4, 5. Highly conserved in evolution, MYC preserves the N-terminal domain (NTD) containing MB1, MB2 for gene transcription regulation and the C-terminal domain (CTD) holding basic helix-loop-helix leucine zipper transcription factors (bHLHLZ) for DNA binding and heterodimerization 4. Acting as a transcription regulator, MYC works with other transcription factors and target genes to regulate many life events like cell growth, apoptosis, metabolism and tumorigenesis 4, 6. Many studies have proven that MYC is a proto-oncogene which has been found deregulated in many cancers including Burkitt's lymphomas, lung carcinoma, breast carcinoma, and colon carcinoma 7-10.

Previous studies have found that MYC is deregulated in 30%-50% of the high-grade breast cancers, whose tumorigenic ability is also confirmed by the MMTV-c-myc/WAP-c-myc transgenic mice 11-14. Notwithstanding all these phenomena we have found, the exact process by which MYC interacts with other genes promoting tumorigenesis remains unknown. Herein, by using online databases, we explored the expression changes, prognosis value, interacting genes, especially the cancer stem cell biomarkers, and the altered signal pathways of MYC during breast cancer tumorigenesis, hoping to shed light on the new biomarkers and therapeutic targets for breast cancer.

## Materials and Methods

### Differential mRNA expression of MYC and its relationship with prognosis

We used ONCOMINE (http://www.oncomine.org), UALCAN (http://ualcan.path.uab.edu), bc-GenExMiner (http://bcgenex.ico.unicancer.fr) and Kaplan-Meier plotter (http://kmplot.com/analysis/) databases to explore the differential mRNA expression of MYC and its relationship with prognosis 61-62. Firstly, we used UALCAN databases to observe the pan-cancer analysis of MYC mRNA expression. Secondly, we used UALCAN and bc-GenExMiner database to explore the differential mRNA expression of MYC in breast cancer subtypes and TNBC subtypes. Finally, Kaplan-Meier plotter was utilized to analyze the interrelation of relapse-free survival (RFS) curves of breast cancer patients with high and low expression of MYC.

### Gene alterations and transcriptional regulations of MYC

We used cBioportal (https://www.cbioportal.org), TRRUST (https://www.grnpedia.org/trrust/) and STRING (https://string-db.org) databases to explore the gene alterations and transcriptional regulations of MYC in breast cancer patients^63^-^65^. In order to obtain the gene alterations in cBioportal, firstly, we used the Breast Invasive Carcinoma (TCGA, PanCancer Atlas) database, then we selected the genomic profiles as Mutations, Putative copy-number alterations from GISTIC, and mRNA expression z-scores relative to all samples (log RNA Seq V2 RSEM). Patient/case set was selected as all samples, and MYC was entered to get the proportion and type of genetic alterations of MYC in breast cancer patients and breast cancer subclasses. To acquire the transcriptional regulations of MYC, we used TRRUST database to get the MYC target genes and transcription factors regulating MYC. Afterwards, data obtained from TRRUST were inputted into the STRING database to obtain the protein interaction network of MYC with its upstream transcription factors and downstream target genes.

### The alternations of signal pathway of MYC and its relationship with cancer stem cells

We used KEGG (https://www.genome.jp/kegg/), TCGA (https://portal.gdc.cancer.gov/), TRRUST (https://www.grnpedia.org/trrust/) and STRING (https://string-db.org) databases to explore the changes of MYC pathway and its relationship with breast cancer stem cells. Firstly, in the KEGG PATHWAY, keyword was entered as c-myc and the name was selected as breast cancer to achieve the pathway alteration map of MYC in breast cancer patients. Secondly, the correlation between MYC and the classic breast cancer stem cells (BCSCs) was analyzed in TCGA database by Pearson's correlation. Then, STRING database was applied to obtain the protein interaction network between MYC and BCSCs. Finally, TRRUST and STRING databases were used to verify the crosstalk between MYC-related transcription factors and classical BCSCs.

### Clinical drug trials involving MYC in breast cancer patients

We utilized the ClinicalTrials (https://clinicaltrials.gov/) database to obtain the clinical drug trial data related to MYC. In ClinicalTrials Database, the status was set as all studies, condition or disease was set as breast cancer, and other terms was set as MYC to obtain the MYC-related clinical drug research data.

## Results

### Interrelation of the changes of the expression of MYC mRNA with the clinicopathological parameters and the clinical prognosis of the breast cancer patients

**Figure 1:**
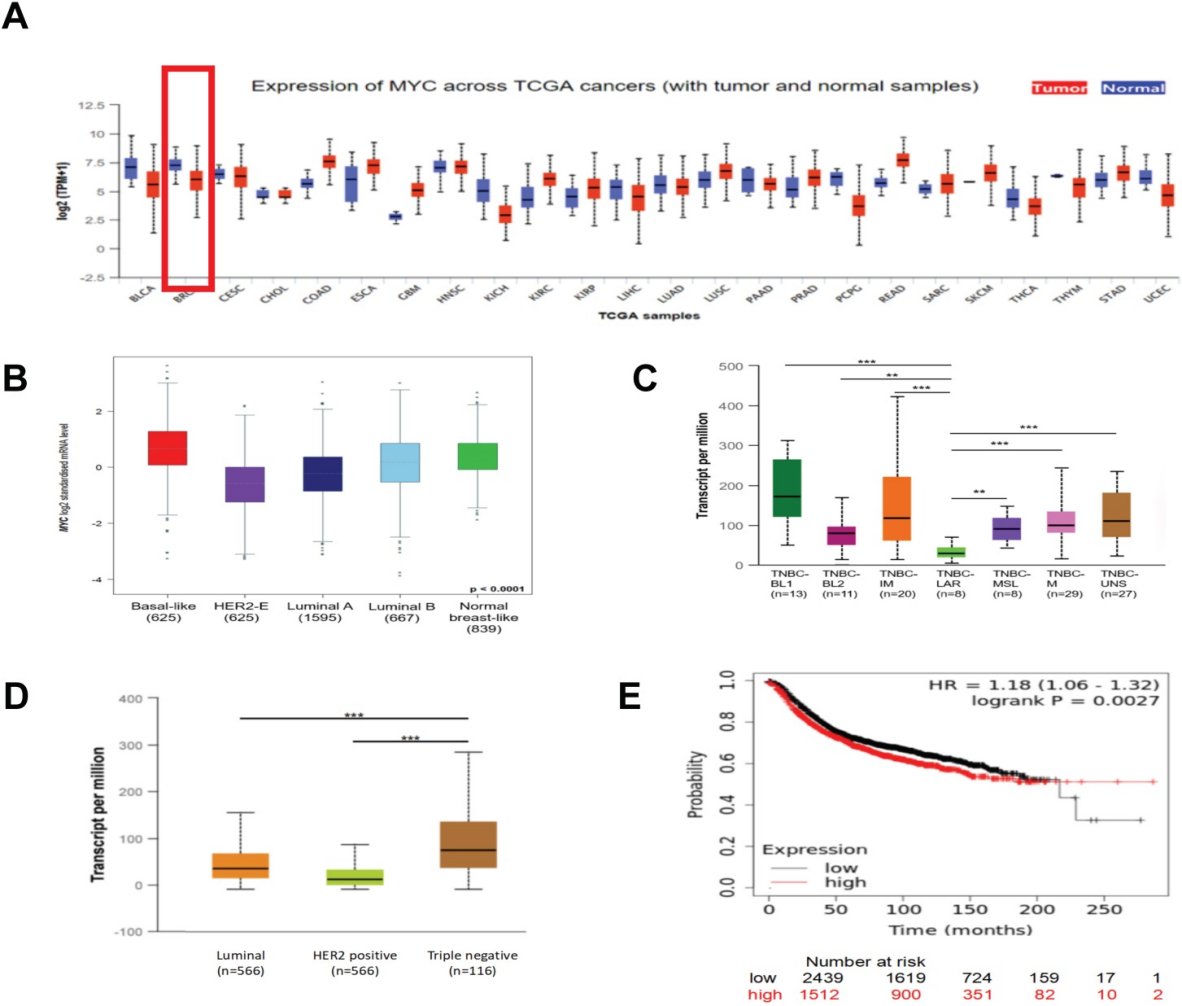
** The expression of MYC and its association with the clinical prognosis of breast cancer patients.** (**A**) The pan-cancer analysis showing the differential expression of MYC in UALCAN database. (**B**) The Box plot depicting the MYC expression analysis according to Sorlie's subtypes in bc-GenExMiner database. (**C**) and (**D**) The Box plots depicting the MYC expression analysis according to TNBC subtypes and breast cancer subclasses, respectively, in UALCAN database. *P< 0.05, **P< 0.01, ***P< 0.001. (**E**) The Kaplan-Meier survival curve of RFS based on MYC expression in breast cancer patients in Kaplan-Meier plotter database (log-rank test, *p*<0.01).

To get an overall idea of the expression of MYC in all tissues and its association with the clinicpathological parameters of the breast cancer patients, we applied UALCAN (http://ualcan.path.uab.edu) and bc-GenExMiner (http://bcgenex.ico.unicancer.fr) databases. According to Figure 1A and 1B, we found that compared with normal breast tissues, the expression of the mRNA of MYC in tumor tissues was decreased expect the basal-like subtype. As was depicted in Figure 1B and 1D, there were significant differences in the expression of MYC among different subtypes of breast cancer, and the expression of MYC was relatively high in TNBC. Furthermore, in the case of the TNBC subclasses, MYC was associated with TNBC-LAR, TNBC-MSL, TNBC-UNS, TNBC-BL2, but showed no statistical difference with TNBC-BL1, TNBC-M, TNBC-IM (Figure 1C). We obtained the prognosis of breast cancer patients corresponding to the expression of MYC by using KMPlot (http://kmplot.cm/analysis) database. As was illustrated in Figure 1E, there was a significant correlation between the expression of MYC and RFS in patients with breast cancer. Nonetheless, the unit that MYC exercises its function is protein. Because of the lack of databases about the relationship between the protein and patient prognosis currently, the association between MYC expression and patient prognosis requires further studies to confirm.

### Molecular mechanisms of the abnormal functions of MYC during breast cancer tumorigenesis

#### Gene alterations of MYC in patients with breast cancer

**Figure 2:**
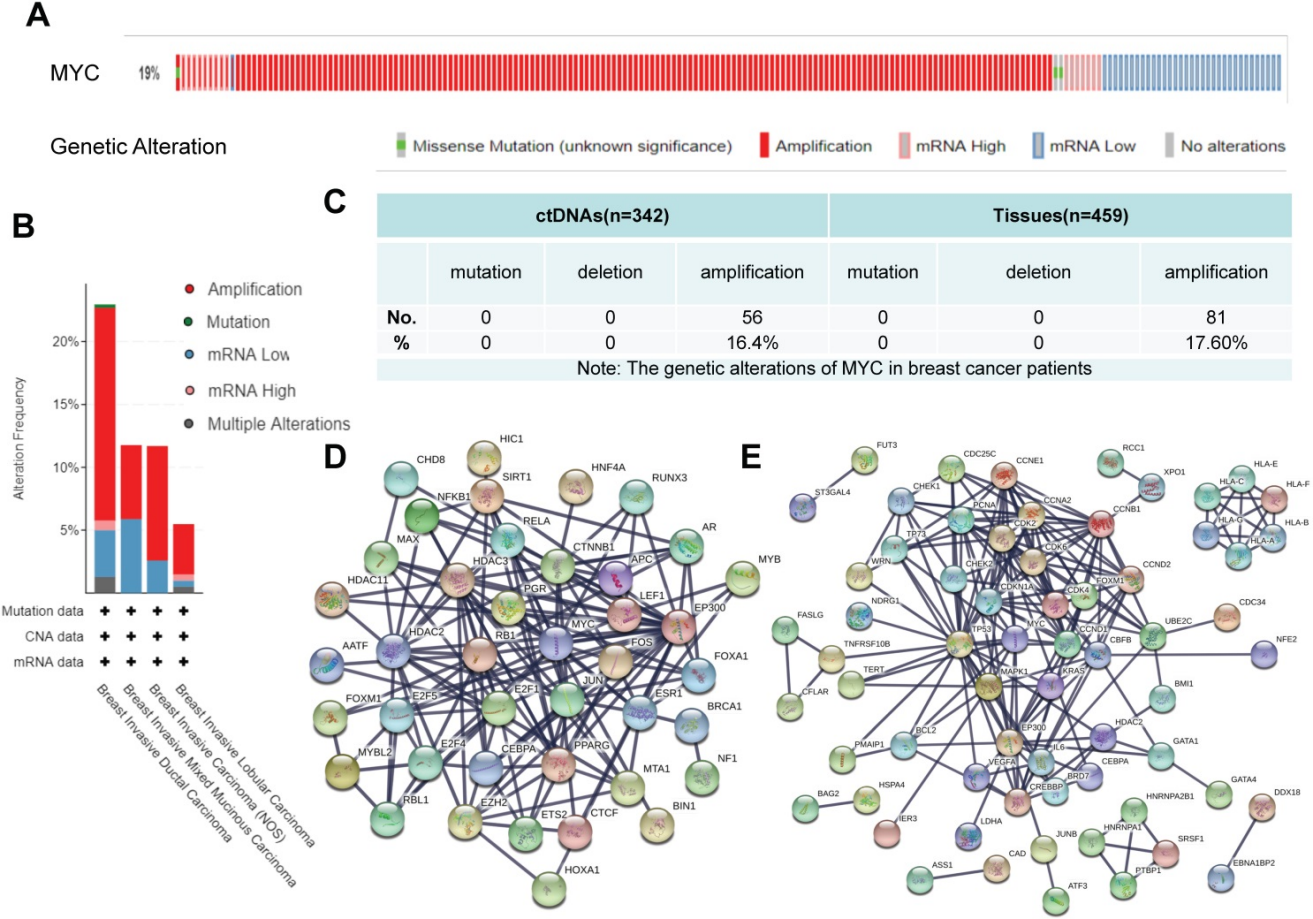
** The genetic alterations and transcriptional regulation of MYC in breast cancer patients.** (**A**) and (**B**) Schematic diagrams depicting the proportion and type of genetic alterations of MYC in breast cancer patients and breast cancer subclasses, respectively, in cBioportal database. (**C**) Sequencing results showing the proportion and type of genetic alterations of MYC in ctDNAs and tissues of breast cancer patients. (**D** and **E**) Protein interaction networks showing the transcription factors regulating MYC and MYC downstream target genes respectively, in STRING database.

In order to explore the gene structure and transcriptional changes of MYC, we used cBioportal (https://www.cbioportal.org), TRRUST (https://www.grnpedia.org/trrust/) and STRING (https://string-db.org) databases. As to what genetic changes have taken place in MYC, according to Figure 2A-2B, the MYC gene was altered in 19% of the patients and amplification was the predominant form of MYC gene alterations in breast cancer. Among the different subtypes of breast cancer, as described in Figure 2B, Breast Invasive Ductal Carcinoma had the highest mutation frequency of MYC, followed by Breast Invasive Mixed Mucinous Carcinoma and Breast Invasive Carcinoma (NOS), whereas Breast Invasive Lobular Carcinoma had the lowest mutation frequency of MYC. In all breast cancer subtypes, however, amplification was the leading form of mutation, accounting for 1/2-3/4 of the frequency. According to our previous data^15^, the frequency of gene alterations of MYC in breast cancer patients was 37.3% from ctDNA and 17.6% from breast cancer tissues (Figure 2C), which was consistent with the results of the database. In order to unveil the transcriptional profile of MYC, we used TRRUST and STRING database to discover the changes in MYC-related transcription factors and obtain the protein interaction network. Protein interaction networks concerning the MYC target genes and the transcription factors regulating MYC were shown in Figure 2D-2E, from which we could find that MYC target genes are closely related to the cell cycle, for example, CDC25C and CDK4/6, and accordingly we could speculate that MYC might be an essential transcription factor regulating the cell growth and proliferation and other life processes. Among the transcription factors regulating MYC, there are many star signal pathway factors, such as SMAD and STAT, which indicates that MYC might also function as an important signal transduction factor.

#### Pathways rewiring of MYC during tumorigenesis

**Figure 3:**
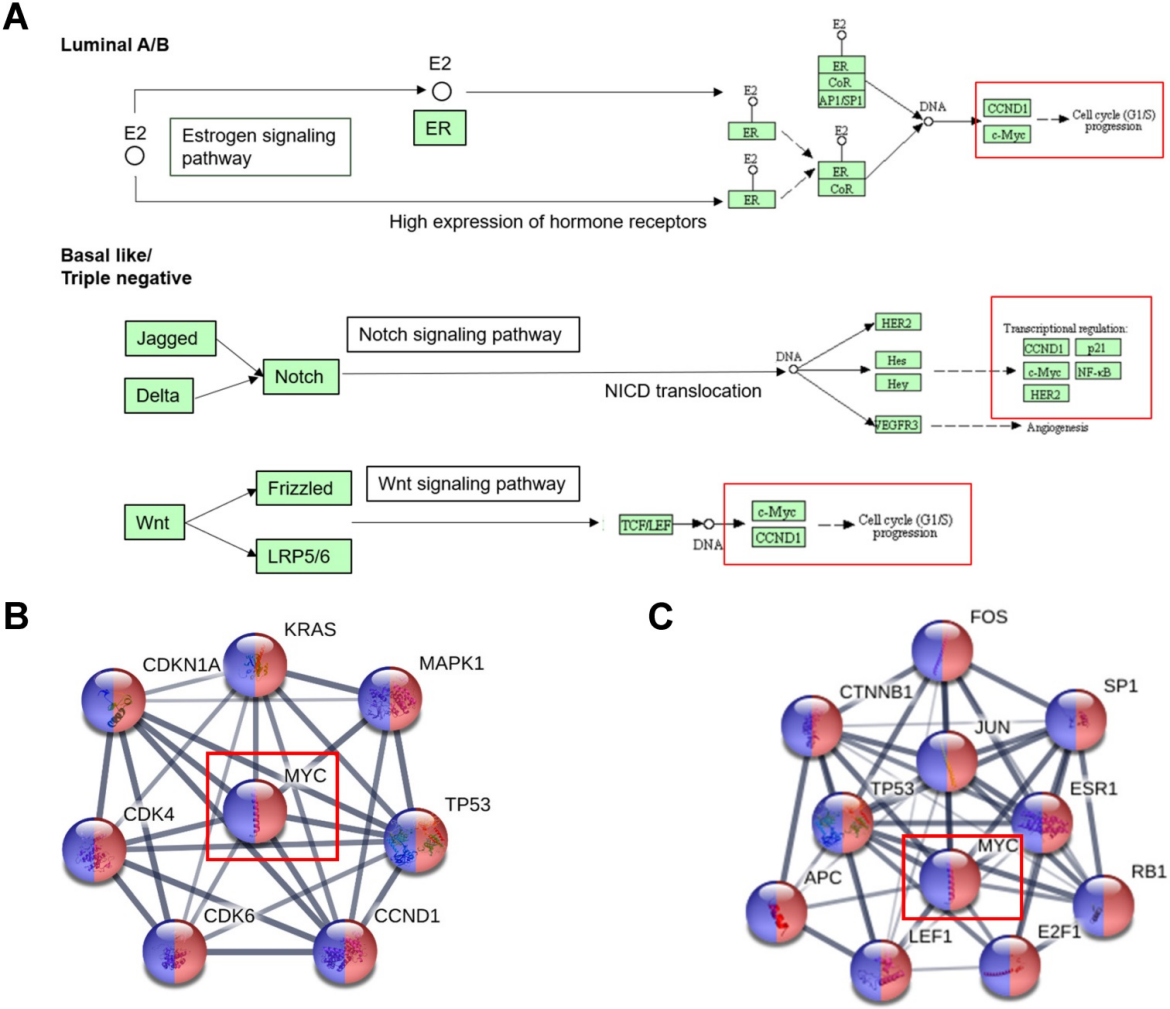
** The alternations of signal pathway of MYC in breast cancer tumorigenesis.** (**A**) Signaling pathways that MYC participates in among different subtypes of breast cancer in KEGG database. (**B** and **C**) Protein interaction networks displaying the transcription factors regulating MYC and MYC targets respectively, under the restrictions of breast cancer (red) and pathways in cancer (blue) in STRING database.

The tumorigenesis can be interpreted as the consequence of an imbalance of the expression of signal pathways. MYC is an important downstream regulator of the signal pathway, so understanding the rewiring pathway of MYC is critical to understand the occurrence of breast cancer. As was shown in Figure 3A, we obtained the signaling pathway changes that MYC participates in among breast cancer subclasses by KEGG database (https://www.genome.jp/kegg/). Based on the figure, we could observe that Luminal A and Luminal B were closely related to hormone-related pathways such as estrogen/progesterone signaling pathway. In addition, basal-like breast cancer was markedly associated with two star signaling pathways of Notch and Wnt. From the chart, we could also ascertain that MYC was dominantly involved in DNA damage repair and cell cycle-related pathways. We could also infer from the figure that MYC did not regulate transcription alone, but interacted with many oncogenes like FGFR1 and PI3KCA and tumor suppressor genes such as p53, PTEN and BRCA1/2 to regulate tumorigenesis, and interacted with many regulatory factors to regulate many essential physiological activities.

In order to further explore the pathway changes of MYC in breast cancer, we applied the MYC transcriptional regulation profile obtained from TRRUST (https://www.grnpedia.org/trrust) database and inputted it into STRING (https://string-db.org) database. Under the restriction conditions of breast cancer and pathways in cancer, we achieved Figure 3B-3C, which displayed the MYC target genes and transcription factors regulating MYC involved in the breast cancer signaling pathway rewiring.

From Figure 3B-3C, we could find that FOS, JUN, E2F1, RB1, ESR1, SP1, APC, LEF1 and CTNNB1 were found in the transcription factors regulating MYC, MAPK1, KRAS, CDKN1A, CDK4/6 and CCND1 were found in the MYC target genes of MYC, and TP53 was present in both the downstream target genes and upstream transcription factors of MYC. All these can provide further clues for the follow-up study of the specific role of MYC in breast cancer pathway remodeling.

#### Correlation between MYC and breast cancer stem cells

A special subgroup of tumor cells, also acknowledged as cancer stem cells (CSCs), can renew itself, as well as promote tumor metastasis, EMT, recurrence and drug resistance 16-20. Because of the heterogeneity, constant dynamic changes, and noteworthy correlation with the shorter OS, DFS in cancer patients, cancer stem cells play an essential role in tumorigenesis 17,18. Here, we use TCGA, STRING database to explore the relationship between MYC and breast cancer stem cells.

**Figure 4:**
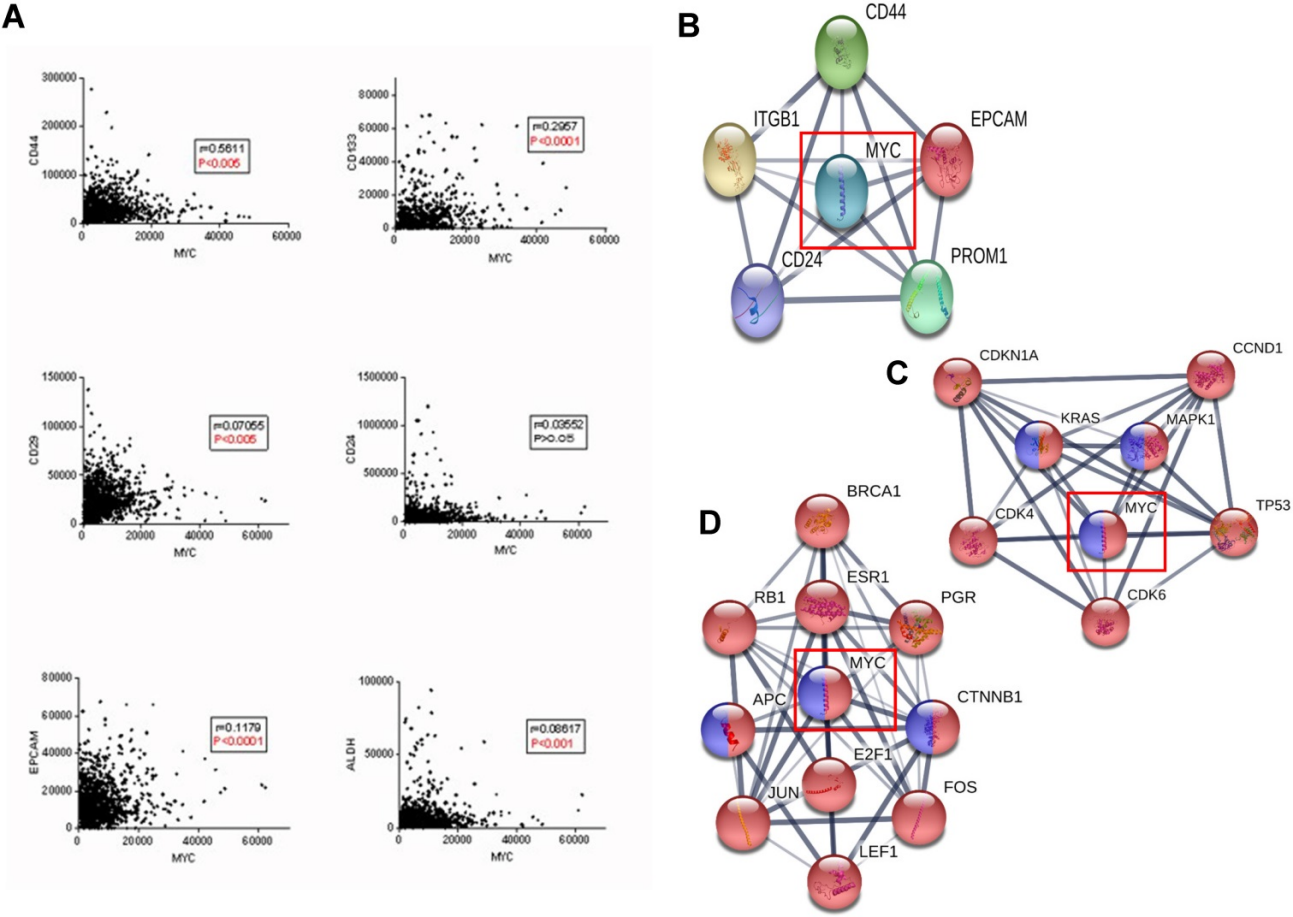
** Relationship between MYC and breast cancer stem cells of the tumor microenvironment during the breast cancer carcinogenesis.** (**A**) Correlations between MYC and breast cancer stem cell markers in TCGA database (Pearson's correlation). (**B**) Protein interaction network between MYC and breast cancer stem cell markers in STRING database. (**C** and **D**) Protein interaction networks showing the transcription factors regulating MYC and MYC targets respectively, under the limitations of breast cancer (red) and signaling pathways regulating pluripotency of stem cells (blue) in STRING database.

According to Figure 4A, MYC was significantly correlated with the classic biomarkers of breast cancer stem cells such as CD44, CD133, CD29, ALDH and EPCAM, but not with CD24, and the protein interaction network between MYC and the biomarkers was depicted in Figure 4B. In order to further verify the relationship between MYC and breast cancer stem cells, we used STRING database, where the KEGG pathways option of analysis section was set to be breast cancer and signaling pathways regulating pluripotency of stem cells, and we found that there is a significant intersection between MYC and the upstream and downstream transcriptional regulators (Figure 4C-D). All these suggested that MYC may regulate the initiation and differentiation of cancer stem cells and therefore, affect the initiation, proliferation and metastasis of breast cancer.

### Advances in the treatment related to MYC

**Figure 5:**
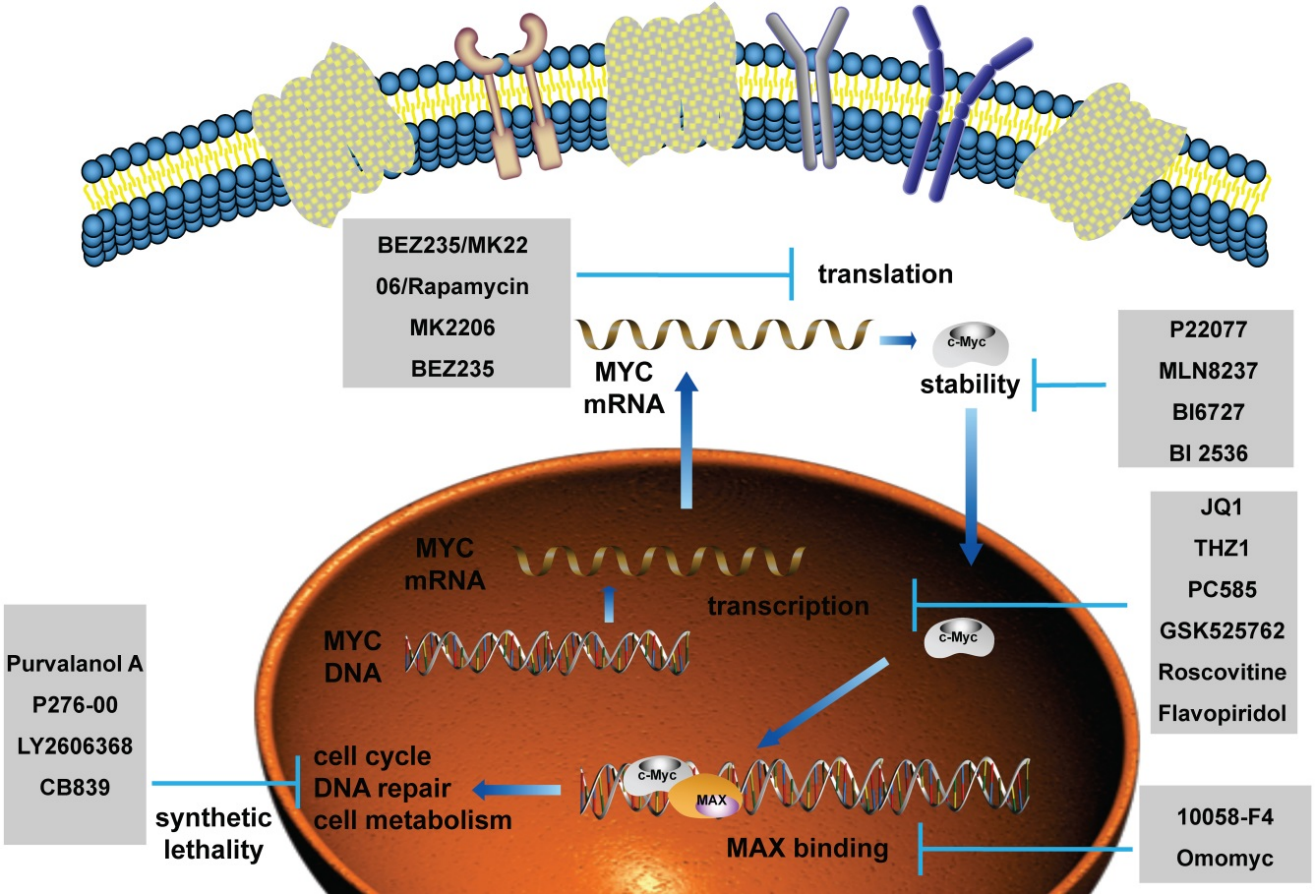
** The current applications of targeted drugs against MYC.** Schematic diagram depicting the therapies and drugs targeting the dysregulated expression of MYC.

As MYC functions as an essential role in promoting tumorigenesis, targeted therapy for MYC has always been a research hotspot. Currently, the treatment of MYC is divided into direct treatment and indirect treatment 21-24. Direct therapy refers to the therapy directly binding to MYC by interfering with its promoter, the recruitment of transcription factors, and the ability to directly bind to other proteins, such as MAX. Among them, the representative drugs are AVI-4126, 10058-F4, Omomyc and so on. Because of the deficiency of an ordered internal structure and a binding capsule of MYC in direct therapy, at present, the researches on MYC treatment are mainly focusing on indirect treatment. Indirect therapy refers to the therapy regulating MYC transcription, translation, stability and synthetic lethality without directly binding to MYC, such as JQ1, P22077, MK2206, and so on 25-33. Pivotal regulatory factors of MYC in these processes and their targeted drugs are summarized in Figure 5. Clinical trials involving MYC in breast cancer patients are summarized in Table 1 using ClinicalTrials database (https://clinicaltrials.gov/).

## Discussion

Conclusively, online database was used to elucidate the alterations of MYC mRNA expression, the relationship between MYC expression and prognosis, the mechanism of MYC deregulation in tumorigenesis and the progress of treatment in breast cancer patients. It was found that the mRNA expression of MYC was decreased in breast cancer patients, which was related to breast cancer subclasses and TNBC subtypes. Moreover, it is the amplification neither mRNA expression nor mutations, that accounted for most alterations of MYC during breast cancer tumorigenesis. Previous studies and our group's sequencing data showed that the amplification rate of MYC in breast cancer patients was approximately 16%-20% 15, 34-36. We also found that the expression of MYC is related to the RFS of breast cancer patients, but the lack of follow-up data of large samples of patients indicates that further large-scale investigation is needed. MYC participated in the vital signal pathway rewiring, including NOTCH, WNT, estrogen and MAPK pathways.

As a transcription factor and proto-oncogene, MYC can not only activate but also inhibit transcription 4, 37. MYC binds to MAX as a heterodimer, recognizing the E-box on the target gene, usually 5'-CACGTG-3' 6, 38-41, and transcription is activated by histone acetylation, chromatin recombination and promoter clearance, and can also be inhibited by identifying the Inr sequence on the target gene 41. MYC regulates a series of processes such as cell cycle, metabolism, apoptosis, and so on. For example, MYC can bind to transcription factors such as TPPAP, CBP and P300 to stimulate acetylation 42, 43, p-TEFb, TFIIH and pol II/III to activate transcription 44, ARP, p53 and caspase 9 to stimulate apoptosis 6, 45. It's the target genes bound by MYC that determine the cellular functions and biological activities.

Recent studies have concentrated on the role and mechanism by which MYC interacts with cancer stem cells. MYC can delay self-degradation by combining with p62, or form a loop with FGF13-AS1 and IGF2BPs to inhibit glycolysis and enhance the stem-like characteristics 18,19. Lee et al. also found that MYC could interact with MCL1 to promoting the drug resistance of TNBC cells by augmenting the mitochondrial oxidative phosphorylated (mtOXPHOS) and reactive oxygen species (ROS) 17. It has been found that MYC is not only connected with the cancer stem cells, but also plays a unique role in the tumor microenvironment. Anna et al. found that MYC interacts with the cancer-associated fibroblasts (CAFs) in the microenvironment participating in the tumor initiation through IGF, IGFBP-6 and other factors 46. Chang et al. found that MYC promotes tumorigenesis by blocking the PI3K/Akt/β-Catenin pathway through the regulation of Slit2/Robo1 47. Recent studies have shown that MYC interacts with macrophages and fibroblasts in the microenvironment to promote tumor brain metastasis and drug resistance by regulating factors such as BrMs, GJA1/Cx43, LIN28B, miR-34a-5p, and so on 20, 48.

MYC is closely correlated with tumorigenesis, such as proliferation, transformation, metastasis, and drug resistance. It regulates cyclin D1, cyclin A, cyclin E and cdc25A which can phosphorylate CDK2/4 6, 49-52, and moreover, it can inhibit p27 and other CKIs 53, 54, collectively modulating DNA synthesis and genomic instability to promote proliferation. MYC can bind to genes related to cell differentiation, such as C/EBP, AP-2 55, 56, and by identifying the internal Inr sequence of these genes which inhibit transcription it can hinder differentiation and promote cell transformation 6, 57. MYC can additionally act as a bridge between cancer stem cell-like properties and metastasis. MYC increases breast cancer stem cell-like properties and EMT by binding to DOT1L and p300, which stimulates SNAIL, ZEB1, ZEB2 transcription and promotes lysine-79 methylation and H3 acetylation through gene epigenetic modification 58. MYC can further enhance the drug resistance by changing tumor cell metabolism which increases glutaminyls and glycolysis 14, 59, 60. Due to the limitation of the sample size of previous studies and lack of follow-up data, the application prospect of MYC as a diagnostic biomarker and prognostic indicator of breast cancer has yet to be further examined. Presently, the therapies targeted MYC are divided into direct and indirect. Due to the shortcomings of the complex internal structure and lack of the binding capsule of MYC, currently, most studies are focusing on the key regulatory factors manipulating the deregulated expression of MYC and the combined therapy with other proto-oncogenes 21-24. More studies should focus on the relationship between MYC amplification frequency and prognosis in large samples of patients, the efficacy of MYC combined targeted therapy and the alterations of specific signal pathway of MYC in the process of breast cancer tumorigenesis.

In conclusion, the differential expression, transcriptional profile, involvement in the cancer pathways alterations, and the interaction with cancer stem cells provide a new idea for targeted molecular therapy of MYC in breast cancer patients and provide clues and directions for a better understanding of the molecular mechanism of breast cancer tumorigenesis.

## Figures and Tables

**Table 1:** Clinical drug trials involving MYC in breast cancer patients

	Trial number	Trial phase	Patients	Interventions	Status
1	NCT00898898	Not Applicable	Primary HER2 positive breast cancer	Other: diagnostic laboratory biomarker analysis	Completed
2	NCT01676753	Phase I	Advanced or Metastatic Breast Cancer; Triple Negative Breast Cancer.	Drugs: Dinaciclib; Pembrolizumab	Active, not recruiting
3	NCT00740532	Not Applicable	Metastatic Breast Cancer	Genetic: Gene mutation analyses and FISH	Completed
4	NCT01534455	Phase II	Metastatic Breast Cancer	Drugs: Lapatinib + 1,23 mg Eribulin; Lapatinib + 1,76 mg Eribulin	Terminated
5	NCT03950570	Phase I	Metastatic Breast Cancer	Drug: Paclitaxel	Recruiting
6	NCT02651844	Not Applicable	Breast Cancer with ratios of PRA/PRB higher than 1.5 and PR higher than 50%.	Drug: Mifepristone	Active, not recruiting
7	NCT01104571	Phase III	Early Breast Cancer	Biological: trastuzumab; Drug: lapatinib ditosylate; Other: laboratory biomarker analysis;Procedures: adjuvant therapy; neoadjuvant therapy; therapeutic conventional surgery.	Active, not recruiting
8	NCT03085368	Phases II & III	HER2-positive Breast Cancer	Drug: lapatinib/trastuzumab	Recruiting
9	NCT00553358	Phase III	HER2/ErbB2 Positive Primary Breast Cancer	Drug: Lapatinib;Biological: Trastuzumab; Drug: Paclitaxel.	The pCR rate was significantly higher in the group given lapatinib and trastuzumab. No significant difference in pCR between the lapatinib and the trastuzumab groups 66.
